# Disseminated Carcinomatosis of Bone Marrow in an African Man with Metastatic Descending Colon Carcinoma

**DOI:** 10.7759/cureus.7593

**Published:** 2020-04-08

**Authors:** Harrison Chuwa, Nadeem M Kassam, Casmir Wambura, Omar A Sherman, Salim Surani

**Affiliations:** 1 Medicine, Aga Khan Hospital, Dar es Salaam, TZA; 2 Internal Medicine, Aga Khan University Medical College, Dar es salaam, TZA; 3 Internal Medicine/Gastroenterology, Aga Khan University, Dar es Salaam, TZA; 4 Internal Medicine/Gastroenterology, Aga Khan Hospital, Dar es Salaam, TZA; 5 Pathology, Aga Khan Hospital, Dar es Salaam, TZA; 6 Internal Medicine, Texas A&M Health Science Center, Bryan, USA; 7 Internal Medicine, Corpus Christi Medical Center, Corpus Christi, USA; 8 Internal Medicine, University of North Texas, Dallas, USA

**Keywords:** dcbm, disseminated carcinomatosis of the bone marrow. crc, colorectal cancer, metastatic cancer, perforated colon

## Abstract

Colorectal cancer (CRC) is ranked third worldwide and seventh in Tanzania. The liver and lungs are the most commonly involved sites. Disseminated carcinomatosis of the bone marrow (DCBM) from colorectal carcinoma is rare and typically indicates widespread disease and poor prognosis. We report a case of a 40-year-old African male, who presented to us with abdominal distension, weight loss, fever and change in bowel habit over the past month. He underwent colonoscopy which revealed a necrotic mass in the descending colon. Biopsies were taken, and histopathology confirmed the presence of poorly differentiated mucin-producing adenocarcinoma. The patient suffered a colonic perforation after the fifth cycle of chemotherapy, requiring surgical interventions. Patient's course was complicated by pancytopenia and bone marrow biopsy revealed infiltration by non-hematopoietic malignant cells and bone marrow necrosis. Disseminated carcinomatosis of the bone marrow is a rare and fatal condition; hence high level of clinical suspicion may help in detecting this rare manifestation of colorectal cancer.

## Introduction

Colorectal cancer (CRC) is the third frequently diagnosed malignancy and fourth leading cause of cancer-related death worldwide, accounting for about 1.4 million new cases and 700,000 deaths in 2012. The incidence of CRC in Tanzania is 462/100,000 (3.1%) with a mortality rate of 323/100,000 (2.8%) [1].

Death in CRC is attributed to the disease recurrence or distant metastases. Distant metastasis in colorectal cancers occurs in liver and lungs [2]. Peritoneum, adrenal glands, bone, spleen and brain are less commonly involved sites [3]. Disseminated carcinomatosis of the bone marrow (DCBM) is a rare condition in which the bone marrow is diffusely invaded with metastases and frequently associated with disseminated intravascular coagulation (DIC) [4]. Both DCBM and DIC are associated with poor prognoses [5].

We hereby report a case of patient with colorectal cancer who developed colonic perforation after his fifth cycle of treatment with folinic acid, 5-fluorouracil and oxaliplatin (FOLFOX-4) together with bevacizumab (BV) (i.e., FOLOX-4 + BV), and subsequently diagnosed with DCBM by bone marrow biopsy after persistent pancytopenia.

## Case presentation

**Figure 1:**
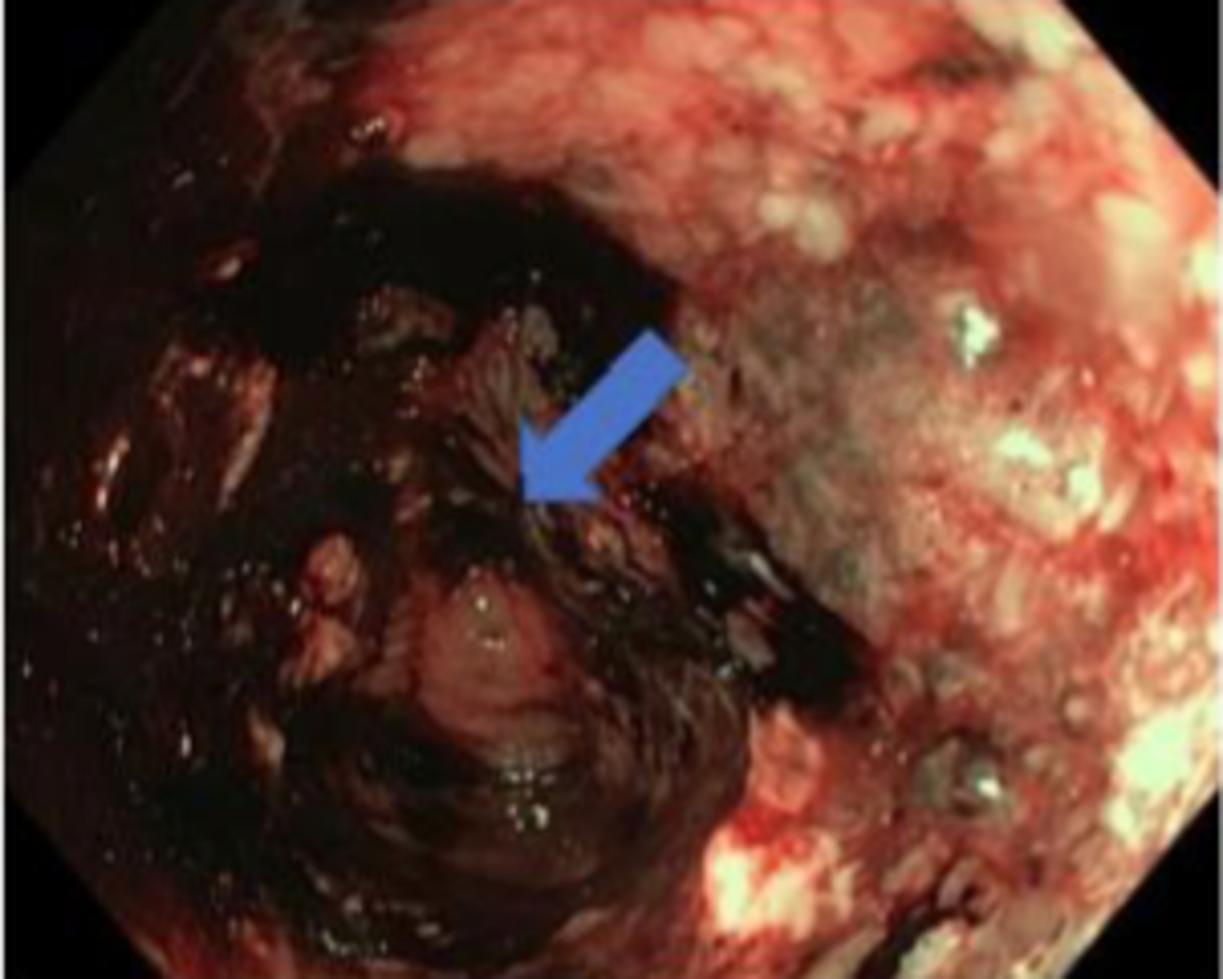
Colonoscopy demonstrating luminal narrowing marked in the descending colon with a necrotic mass which easily bleeds on contact.

A 40-year-old African male presented to our center with the complaint of bloody diarrhea, accompanied with progressive abdominal distension for one month. The colonoscopy revealed luminal narrowing of the descending colon with a necrotic mass at the descending colon which was edematous and bled with minimal contact (Figure 1).

**Figure 2:**
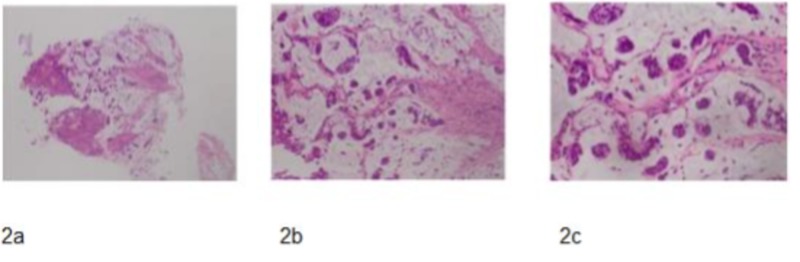
(a) H&E – X4 Objective – epithelial tumor cells floating within lakes of mucin. (b) H&E – X10 Objective – malignant epithelial cells floating within lakes of mucin. (c) H&E – X20 Objective – malignant epithelial cells exhibiting high nuclear to cytoplasmic ration, irregular nuclear contours, nuclear hyperchromasia and cytoplasmic mucin which in some cells are seen to indent the nuclei - background mucin is evident.

The histopathology revealed poorly differentiated mucin-producing adenocarcinoma (Figure 2a, 2b, 2c).

Abdominal computed tomography (CT) scan revealed liver metastases with peritoneal seedlings. The patient was started on palliative chemotherapy FOLFOX-4 + BV and was followed up on outpatient basis.

**Figure 3:**
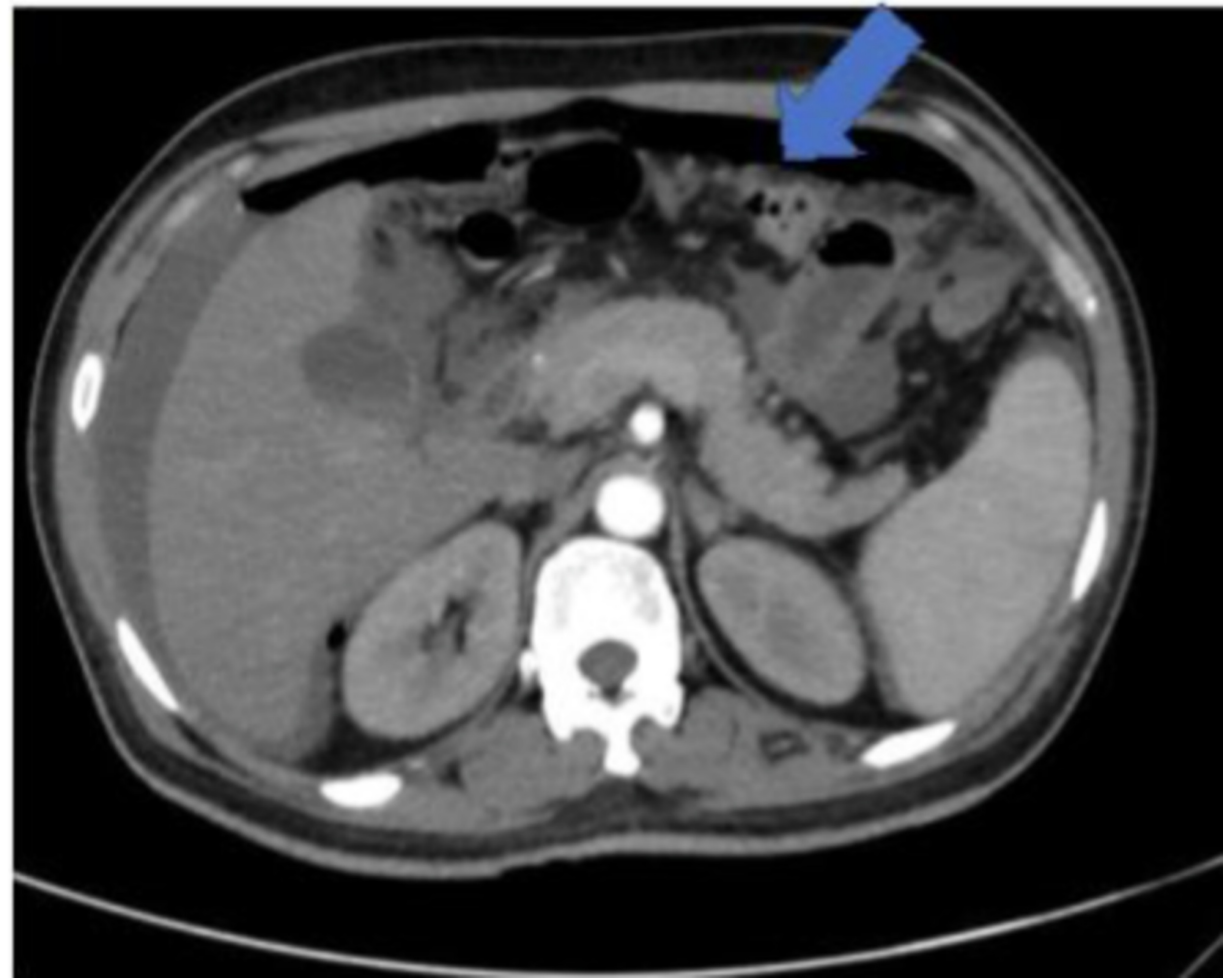
Abdominal CT scan revealing pneumoperitoneum and intraperitoneal fluid secondary to bowel perforation.

However, five days after the fifth cycle of chemotherapy, the patient presented to the emergency department with severe acute generalized abdominal pain, progressively increasing, worsened by movement accompanied with abdominal distension and constipation. No nausea, fever or vomiting was reported. The patient was mildly dehydrated, with the sodium of 150 meq/L, and distended abdomen. The rest of the physical and laboratory examinations were unremarkable except for white blood cell count of 13.8 x 10^9^. CT examination of the abdomen was performed, which revealed bowel perforation (Figure 3) and the patient was started on conservative treatment with the double lumen nasogastric tube (NGT) together to help with abdominal distension. The patient was also started empirically on antibiotics (cefepime and metronidazole).

By fifth day of conservative treatment, the patient had developed severe generalized tenderness and had to undergo exploratory laparotomy, which showed matted bowel, thickened omentum, turbid fluid with feculent matter in left paracolic area and tumor in the descending colon, stuck to posterior abdominal wall with a large perforation. Subsequently, the patient underwent loop ileostomy.

The patient's post-surgical course was complicated by pancytopenia, with white blood cells 0.7 x 10^9^/L; hemoglobin 8 g/dL; and platelets 70 x 10^9^/L. Peripheral blood film (PBF) revealed normocytic, normochromic erythrocytes, leucopenia with no blast cells.

**Figure 4:**
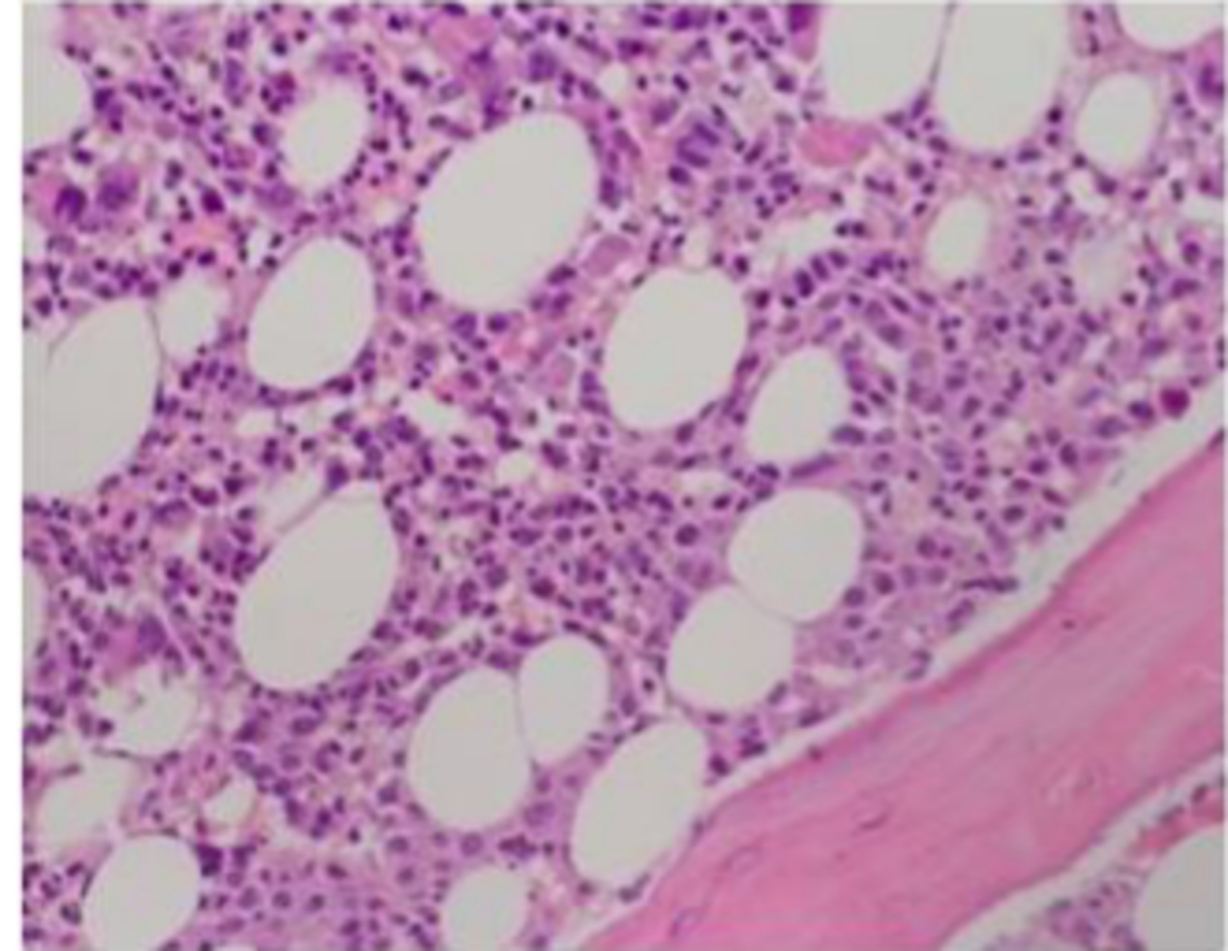
H&E – X20 Objective – Hypercellular marrow with infiltration of clusters of non-haemopoietic cells - some dispersed within marrow storma.

Bone marrow biopsy revealed bone marrow infiltration by non-hematopoietic malignant cells and bone marrow necrosis, i.e., disseminated carcinomatosis of bone marrow (DCBM) (Figure 4).

The patient’s condition got worse, he went into multi-organ system failure, and expired as an inpatient.

## Discussion

This case report represents the first documented case of DCBM of a CRC patient in Tanzania and the first case in an African race.

DCBM with DIC is an oncological emergency which unfortunately is very difficult to diagnose when a patient is alive and able to tolerate chemotherapy [6]. Bone marrow metastasis from the solid tumor is seen in patients with lung, breast and prostate cancer, though is very rare in patient with colon cancer [7,8]. Micro metastasis of the bone marrow occurs in over one-third of the patients with stage I-III colon cancer and is associated with poor prognostic factor for overall survival and disease-free survival [9]. Our patient tolerated five cycles of chemotherapy (FOLFOX-4 + BV) but died due to complications of bowel perforation.

Although most reported cases of DCBM secondary to CRC are associated with DIC, this case had no DIC until towards end [4]. In our case, the DCBM impeded the patient’s recovery from surgery and was diagnosed late due to low suspicious index of DCBM in CRC.

## Conclusions

DCBM should be suspected in patients with CRC with abnormal complete blood count (CBC) results or DIC. Prompt bone marrow biopsy should be performed to establish a definitive diagnosis. Early diagnosis of DCBM is important. Clinicians need to watch out for DIC and counsel the patient and family on the poor prognosis associated with this disease entity.
